# The zebrafish Multivariate Concentric Square Field: A Standardized Test for Behavioral Profiling of Zebrafish (*Danio rerio*)

**DOI:** 10.3389/fnbeh.2022.744533

**Published:** 2022-03-17

**Authors:** Laura E. Vossen, Ronja Brunberg, Pontus Rådén, Svante Winberg, Erika Roman

**Affiliations:** ^1^Division of Anatomy and Physiology, Department of Anatomy, Physiology and Biochemistry, Swedish University of Agricultural Sciences, Uppsala, Sweden; ^2^Neuropharmacology, Addiction and Behavior, Department of Pharmaceutical Biosciences, Uppsala University, Uppsala, Sweden; ^3^Behavioral Neuroendocrinology, Department of Neuroscience, Uppsala University, Uppsala, Sweden; ^4^Behavioral Neuroendocrinology, Department of Medical Cell Biology, Uppsala University, Uppsala, Sweden

**Keywords:** anxiety-related behavior, behavioral test, explorative behavior, locomotory activity, novel tank diving test, risk-taking

## Abstract

The zebrafish (*Danio rerio*) is an important model organism in the study of the neurobiological basis of human mental disorders. Yet the utility of this species is limited by the quality of the phenotypical characterization tools available. Here, we present a complex testing environment for the quantification of explorative behavior in adult zebrafish, the zebrafish Multivariate Concentric Square Field™ (zMCSF), adapted from the rodent equivalent that has been used in > 40 studies. The apparatus consists of a central open area which is surrounded by a dark corner with a roof (DCR), corridors, and an inclined ramp. These areas differ in illumination, water depth, and are sheltered or exposed to different degrees. We quantified behavior of male and female wild-caught and AB strain zebrafish in the zMCSF (day 1) and cross-validated these results using the novel tank diving test (NTDT) (day 2). To assess the effect of repeated testing, AB zebrafish we tested a second time in both tests 1 week later (on days 7 and 8). We detected strong differences between the strains, with wild zebrafish swimming faster and spending more time in the corridors and on the ramp, while they avoided the open area in the center. AB zebrafish were less hesitant to enter the center but avoided the ramp, and often left one or more zones unexplored. No major sex differences in exploratory behavior were detected in either strain, except for a slightly higher velocity of AB males which has been reported before. Importantly, the zMCSF was largely resilient to repeated testing. The diving test revealed only one difference confined to one sex; wild females paid more visits to the top third than AB females. In isolation, this finding could lead to the conclusion that wild zebrafish are more risk-taking, which is incorrect given this strain’s avoidance of open areas. To conclude, our results suggest that the zMCSF presents a sophisticated behavioral tool that can distinguish between different magnitudes and types of risk, allowing the user to create an intricate behavioral profile of individual adult zebrafish.

## Introduction

Knowledge of the local environment can entail important advantages for animals, both in terms of survival and reproduction. When placed in an unfamiliar environment, animals strategically explore their novel surroundings to locate food, water and hiding places, and to assess whether conspecific competitors or predators are present (Winkler and Leisler, 1999). In many species ranging from rats to ants, exploratory behavior is structured around a familiar “home base,” a location (often close to a wall or corner) in which the animal spends a disproportional amount of time and from which it makes round trips in different directions (Eilam and Golani, 1989; Collett and Zeil, 2018). If the animal encounters novel objects or structures these may be investigated, manipulated or avoided (Belzung and Lepape, 1994). Defensive behaviors such as hiding and freezing (Walsh and Cummins, 1976) and scanning head movements (Dingemanse et al., 2002) occur more readily in a novel environment.

Certain aspects of exploratory behavior can be measured using classical behavioral tests (Hanell and Marklund, 2014; Stewart et al., 2014), including home base behavior (Eilam and Golani, 1989; Stewart et al., 2010) and avoidance of brightly illuminated open spaces or elevated platforms (Rodgers, 1997). Other aspects remain concealed by the simplicity of the apparatus’ design. First, limited physical structure means that the whole apparatus can be overseen from one or more positions, which reduces the appropriateness of flight and risk assessment behaviors such as scanning head movements or “corner runs” [fast movement from one shelter to the next through an exposed space (Blanchard and Blanchard, 1989)]. In such an environment there is furthermore less novelty, which is rewarding in itself (Kakade and Dayan, 2002) and provides an incentive for exploration. Second, in classical tests the decision between safety and exploration is often binary (e.g., wall vs. center, open vs. closed arm), and the animal’s choice away from safety automatically assumes a choice for exploration. This while shelter seeking, roaming around in an area of relative safety, and actively seeking novelty may be considered different choices. Indeed, classical tests often represent only one type of risk (Hanell and Marklund, 2014; Stewart et al., 2014), whereas in nature the animal may need to balance risks of different kind and magnitude against each other. A final drawback of many classical tests is that animals often habituate to the novelty offered in the same test (Rodgers, 1997) and even generalize experiences from one test to another in a test battery (McIlwain et al., 2001; Blokland et al., 2012). Therefore, classical tests are less suitable for experimental designs that involve repeated testing of the same individuals. Taken together, the above considerations led to the development of a novel test apparatus for rodents, the Multivariate Concentric Square Field™ (MCSF; Meyerson et al., 2006; Roman and Colombo, 2009).

The zebrafish (*Danio rerio*) continues to increase in popularity as a model organism (Kalueff et al., 2014; Gerlai, 2020). Classical behavioral tests have already been translated to this species, including the open field (Stewart et al., 2012), light/dark (Maximino et al., 2010), and plus maze (Varga et al., 2018) tests, in addition to the highly used novel tank diving test (NTDT) (Levin et al., 2007). In the current study we adapted the standardized MCSF arena for rodent behavioral profiling, the MCSF, to zebrafish (zMCSF) (Bikovski et al., 2020). The apparatus consists of a central open area which is surrounded by a dark corner with a roof (DCR), corridors, and an inclined ramp. These areas differ in illumination and water depth, are sheltered or exposed to different degrees, and the arena cannot be overseen from any of these areas. This design offers the fish a free choice between several alternative locations of different quality in terms of risk and safety, while also providing an incentive for exploration. This generates a comprehensive and detailed behavioral profile of an individual zebrafish within a single behavioral test (Bikovski et al., 2020), while avoiding carry-over effects common to many test batteries (McIlwain et al., 2001).

In rodents, the sheltered dark corner room (DCR) is considered a safe and the elevated and illuminated bridge a risky area, based on the observations from pup retrieval, food hoarding and shelter seeking behaviors (Meyerson et al., 2006). The most central area of the MCSF, i.e., the central circle, is avoided by rats and they pass it at greater speed than neighboring areas (Meyerson et al., 2006). Moreover, administration of the benzodiazepine diazepam or alcohol increases the duration of visits to the MCSF center (Meyerson et al., 2013; Karlsson and Roman, 2016), suggesting that also this area is perceived as relatively risky, similar to the center of an open field (Momeni et al., 2014). In areas leading up to the elevated and illuminated bridge (slope and bridge entrance) stretched attend postures are most often observed (Augustsson and Meyerson, 2004; Meyerson et al., 2013), therefore these areas have been suggested as areas for risk assessment (Macintosh and Grant, 1963; Rodgers et al., 1999). Finally, the corridors act as semi-sheltered transit zones for entering the different areas of the arena (Augustsson and Meyerson, 2004; Roman and Colombo, 2009). In several rodent studies published so far, the MCSF has been used alongside classical tests such as the open field or elevated plus maze. This has not only provided important cross-validation, the MCSF has also repeatedly picked up on effects that were not registered by traditional tests (Birgner et al., 2010; Roman et al., 2012), such as the effect of low doses of the benzodiazepine diazepam not detected in the elevated plus maze (Meyerson et al., 2013).

The zMCSF has previously been optimized regarding the dimensions of the arena (Roman et al., 2016, 2018; Bikovski et al., 2020). Herein we optimized the zone settings and highlight strain differences between AB and wild zebrafish, test for sex differences within strains and report the effect of repeated testing in AB zebrafish. We also tested the same individuals in the NTDT, which gives further clues to the interpretation of the zones in the zMCSF. We compare exploratory behavior in the zMCSF to published studies that used classical tests to measure the effects of repeated testing, strain and sex. Finally, we evaluate similarities and differences between how zebrafish, mice and rat behave in the MCSF, and ask whether laboratory animals of these species show similar differences in explorative behavior and behavioral profiles.

## Materials and Methods

### Animals and Housing

Experiments took place at the Department of Neuroscience, located at the Biomedical Center, Uppsala University in Sweden in September and October 2017. Ethical approval for the use of animals was given by the Uppsala Regional Animal Ethical Committee (permit C55/13), following the guidelines of the Swedish Legislation on Animal Experimentation (Animal Welfare Act SFS1998:56) and the European Union Directive on the Protection of Animals Used for Scientific Purposes (Directive 2010/63/EU).

In the current study, a total of 73 zebrafish were involved in behavioral testing; 13 females and 17 males of the AB strain, and 21 females and 22 males of the “wild” strain, i.e., offspring of wild-caught fish. Adult AB zebrafish (d.o.b. October 2015) were obtained from SciLifeLab (Evolutionary Biology Centre, Uppsala University) and transferred to the Department of Neuroscience. The wild-caught strain was collected from the river Ichamati (approximately 70 km from Calcutta), bred in ponds, and the resulting offspring were transferred as adults to the Department of Neuroscience where they were kept in two large, aerated aquaria (200 L). The lab-raised F1 offspring (d.o.b. April 2016) from the wild-caught individuals were included in this experiment, and will hereafter be referred to as “wild zebrafish.” Hence all animals were adults at the time of testing; the AB zebrafish were 23 months and the wild strain was 18 months of age.

Experimental animals were kept in 9.5L tanks in a stand-alone rack system (Aquaneering, San Diego, United States) that was maintained at 27 ± 1.5°C with a photoperiod of 14L:10D (lights on at 07:00 AM). The aquarium system contained a particular filter pad (exchanged weekly), a fluidized bed biological filter, carbon filters and an ultraviolet sterilizer. Fish tanks were supplied with recirculating copper-free Uppsala municipal tap water of which 10% was exchanged daily. Alkalinity (66–119, median 87 mmol L^–1^), conductivity (34–48, median 40 mS m^–1^) and pH (8.2–8.5, median 8.4) were monitored daily. Animals were fed twice daily with flakes (tropical energy food, Aquatic Nature, Roeslare, Belgium) and *Artemia* brine shrimp (Argentemia Platinum Grade 0, Argent Aquaculture, Redmond, United States).

#### Visual Implant Elastomer Tagging

Two weeks prior to the first behavioral test, AB zebrafish were anesthetized with tricaine (Sigma-Aldrich, Sweden) and injected with a visual implant elastomer (VIE) tag (Northwest Marine Technology, Anacortes, United States) using two colors at four possible tagging positions (Hohn and Petrie-Hanson, 2013). After recovery from the anesthetic, animals were placed in same sex groups of 6–12 individuals in 2.8 L tanks, allowing for unique tags within each tank and creating smaller groups that could be tested within one session. The wild zebrafish were not tagged but were also placed in small same sex groups.

### Behavioral Test Procedures

AB zebrafish were tested twice in the zMCSF test (on experimental days 1 and 7; Run 1 and 2, respectively), and twice in the NTDT (on days 2 and 8; Run 1 and 2, respectively). Wild zebrafish were tested once in each test (zMCSF on day 1 and NTDT on day 2). The morning feed was provided at least 30 min before behavioral tests were performed. None of the zebrafish used in this experiment had any previous experience of behavioral testing. All behavioral tests took place in a separate room located inside the aquarium room. The experimenter was not present or visible during video recordings. Male groups were tested before female groups, to minimize confounding effects of pheromones from ovulating females. Between trials, testing arenas were sprayed with ethanol (96%), rinsed twice with water and refilled.

#### The zebrafish Multivariate Concentric Square Field Test

**FIGURE 1:**
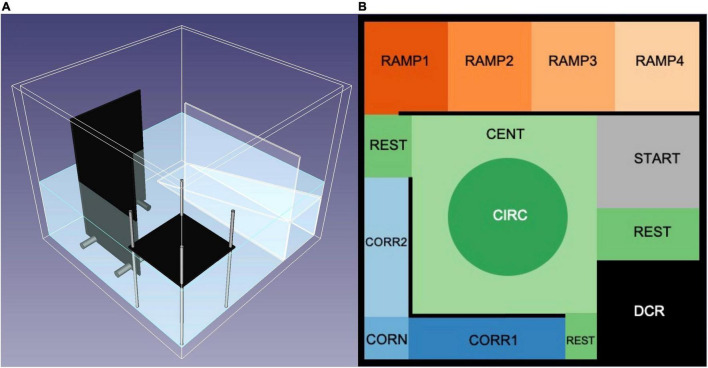
The zMCSF testing arena. **(A)** 3D model of the zMCSF arena, created with FreeCAD software (Riegel et al., 2017). The zMCSF contains a dark corner roof (DCR), two walls building a corridor and corner, and an inclined ramp creating decreasing water depth, all of which surround a central open area. For exact measurements see Supplementary Material. **(B)** Virtual division of zones in the arena, as seen from above, used for tracking in Ethovision XT15 (Noldus, Wageningen, Netherlands).

The zMCSF consisted of a square aquarium (30 × 30 × 25.8 cm, water depth 10 cm) filled with 8 L pre-heated copper-free Uppsala municipal tap water (23 ± 2°C) and containing three objects; a roof, a corridor and a ramp, which were placed around the walls thereby surrounding a central open area (Figure 1A, blueprints provided in Supplementary Figure 1). These objects created 12 zones (Figure 1B): a dark corner with a roof (DCR), a semi-sheltered area consisting of two corridors (CORR1 and CORR2) and a corner (CORN), an inclined ramp leading from high to low water depth (RAMP1-4), a central square consisting of an central circle (CIRC) and the remnant of the central square (CENT) and finally the remaining floor area that did not belong to any of the other zones (REST). An infrared backlight (Noldus, Wageningen, the Netherlands) was placed under the zMCSF arena and an infrared camera (JVC SuperLoLux, Yokohama, Japan) on the ceiling to record the movement of the fish in the arena. The aquarium was made out of 7.5 mm thick transparent Perspex while the ramp consisted of 2 mm transparent perspex. The roof and corridors were composed of infrared transparent plastic, which appears untransparent to the fish but enables infrared video recording of the animal in these areas. Stainless steel pillars kept the DCR and corridors in place. Two photographic lights (Walimex daylight 1000, the Hague, the Netherlands) provided ambient lighting of 0.46 Lux (Lux meter, Fisher Scientific Ltd., Uppsala, Sweden).

Zebrafish were netted out of their 2.8 L housing tank, transferred in a 500 mL beaker, and released in the zMCSF arena at the START zone (Figure 1B). Videos were recorded with Ethovision XT12 (Noldus, Wageningen, the Netherlands), with recording starting 2 s after the fish was detected in the arena and lasting for 30 min. Thereafter, the fish was transferred to a 1.8 L tank where it was kept until all fish from its home tank were tested, and the group was reunited in the home tank.

Using Ethovision XT15 (Noldus, Wageningen, the Netherlands) we manually assessed all tracks for any tracking errors caused by reflections and extracted six variables from the videos. For the whole arena we extracted duration in arena (only used to check data integrity), total distance moved (cm) and average velocity (cm s^–1^). For each zone, we extracted the cumulative duration (s) in zone, frequency of zone visits and latency (s) until first entry into zone. For the REST zone only the duration (s) in this zone could be extracted. From these variables, we derived five more ethologically relevant variables, following analyses of MCSF tests in rodents (Augustsson and Meyerson, 2004; Meyerson et al., 2013). Total activity (abbreviated as “Totact”) was calculated as the sum of all zone frequencies (entries). Average duration per visit (s) was calculated as the total duration in zone divided by the frequency of visits to that zone. Frequency (%), the percentage of visits to a zone, was computed as the frequency of visits to that zone divided by total activity. The zone in which the fish spent the longest cumulative duration was defined as the individual’s home base (Eilam and Golani, 1989). Using the latency (s) variable, we derived the number of zones entered by the fish, and if the fish had explored all zones (“fully explored,” a binary variable). Finally, these same variables were extracted from Ethovision in “minute bins,” allowing for analyses over time.

#### The Novel Tank Diving Test

We followed the original description of the NTDT (Levin et al., 2007), with minor modifications. Video recordings were made using the same equipment as outlined in section “The zebrafish Multivariate Concentric Square Field Test,” now placing the infrared light board behind the arena and filming the arenas from the side. We used a 1.8 L zebrafish housing system tank as an arena (LxWxD 26 × 5 × 12 cm; ZT180, Aquaneering, San Diego, United States) filled with 1.75 L pre-heated copper-free Uppsala municipal tap water (23 ± 2°C). The arena was horizontally divided into three zones of equal height (4 cm): the bottom (BOT), middle (MID), and top zone (TOP). An open shelving system (IVAR, IKEA, Sweden) allowed for simultaneous recording of up to eight arenas. The short sides of each tank were covered with white plastic film to prevent the fish from seeing into the neighboring tank. Each NTDT trial lasted 15 min, after which the fish was placed back in its home tank. We extracted the same variables from the video tracking software as described for the zMCSF (see section “The zebrafish Multivariate Concentric Square Field Test”).

### Statistical Analyses

All statistical analyses were carried out in R statistical computing software version 4.0.2 (R_Core_Team, 2020) with added packages “lmer” (Bates et al., 2015), “emmeans” (Lenth, 2020), “bestNormalize” (Peterson and Cavanaugh, 2019), “ggplot2” (Wickham, 2016), “pals” (Wright, 2019) and “ggalluvial” (Brunson and Read, 2020). The data was split up into two datasets, since the experimental design was not fully factorial (i.e., there was no Run 2 for the wild zebrafish). The “retested dataset” contained the data from all tests on the AB zebrafish, while the “strain dataset” contained the data from both strains on the first testing occasion. We first explored the data by conducting a principal component analysis (PCA) on each dataset, using the “prcomp” function with scaling and centering of variables.

#### Strain and Sex Differences

To evaluate the effect of Strain and Sex on total distance moved (cm) and mean velocity (cm s^–1^), we computed two-way ANOVAs with main effects of Strain (AB or wild) and Sex plus the interaction effect. Total activity was modeled with a generalized linear model (GLM) with a negative binomial error distribution, using the same explanatory variables. For zone specific variables total duration (s), average duration per visit (s) and latency (s) a linear mixed-effects model (LMM) was constructed with fixed effects of Zone, Strain, Run and Sex and their interactions and a random intercept of Individual. Latency was transformed using an ordered quantile normalization (“orderNorm” function), a rank-based procedure, suggested by the “bestNormalize” function (both functions are part of the “bestNormalize” package). For count variables frequency and frequency (%) we computed a negative binomial generalized linear mixed-effects model (GLMM), with the same fixed and random effects, after a Poisson GLMM proved to suffer from overdispersion. For all models, *post hoc* pairwise comparisons with Bonferroni correction for multiple testing were computed using the “emmeans” function.

To evaluate the home base behavior of AB vs. wild zebrafish, we constructed a Poisson GLM with the number of zones explored as a response variable and Strain, Sex and their interaction as explanatory variables. In addition, a binomial GLM with the same explanatory variables was performed on the binary variable indicating whether all zones had been visited.

#### Effect of Repeated Testing

For arena wide variables total distance moved (cm) and mean velocity (cm s^–1^) in the retesting dataset, we constructed two linear mixed effects models (LMM) with fixed effects of Run (1 or 2) and Sex (female or male) and their interaction, and a random intercept of Individual. Total activity was modeled using a GLMM with negative binomial error distribution with the same explanatory variables. To test for an effect of retesting on zone specific variables total duration (s), average duration per visit (s), frequency, frequency (%) and latency (s) we constructed similar (G)LMMs as described in section “Strain and Sex Differences,” but the fixed effect of Strain was exchanged for a fixed effect of Run.

We calculated consistency repeatability between Run 1 and 2 as the intraclass Pearson’s correlation coefficient (ICC) per zone, variable and group (Sokal and Rohlf, 1995; Nakagawa and Schielzeth, 2010) using the “cor.test” function. Total duration (s) and average duration per visit (s) were log transformed while for variable latency (s) an ordered quantile normalizing transformation was applied (orderNorm’ function). To assess the relationships between zones (e.g., did individuals that spent more time in the DCR also spent less time in the RAMP?), we created five correlation matrices per Strain and Run (i.e., AB Run 1, AB Run 2 or wild zebrafish), one for each of the five zone-related variables, using the “chart.Correlation” function from the package “PerformanceAnalytics” with logarithmic transformations of the same variables as for the repeatability correlations.

To assess whether more zones were explored in the second run, we constructed a generalized linear model with Poisson error distribution (Poisson GLM) using the number of zones explored as a response variable and Run, Sex and their interaction as explanatory variables. Finally, a binomial GLM with the same explanatory variables was performed on the fully explored variable (binary variable).

#### Behavior in the Novel Tank Diving Test

The NTDT variables were assessed for effects of Strain, Sex and Run in the same fashion as the zMCSF variables, as described in sections “Strain and Sex Differences” and “Effect of Repeated Testing” Home base behavior was not assessed in the NTDT, since most animals spent the longest duration in the bottom zone.

## Results

### Principal Component Analysis

**FIGURE 2:**
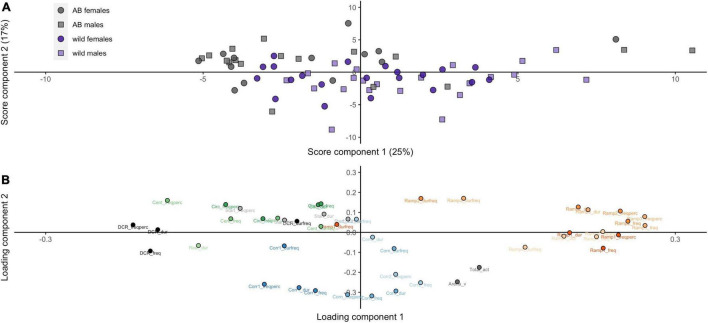
Principal component analysis (PCA) of the behavior from the zMCSF for wild and AB zebrafish (for AB zebrafish, only Run 1 is included here). Scatterplots of **(A)** individual scores on PC2 and PC1 (score plot) and **(B)** variable loadings on PC2 and PC1 (loading plot). CENT, center; CIRC, central circle; CORN, corner; CORR, corridor; DCR, dark corner roof; REST, the part of the arena not designated to any other zone; Dur, duration in zone (s); Durfreq, average duration per visit (s); Freq, frequency; Freqperc, frequency divided by the total number of zone entries to all zones (%); V, mean velocity in the arena (cm s^–1^).

Our initial PCA on the variables from the zMCSF revealed largely overlapping distributions of the two sexes within the same strain, while AB fish only partially overlapped with the wild strain (Figure 2A). The loading plot showed a clear separation of test variables (Figure 2B). The DCR, the CENT/CIRC and the RAMP variables all fell on the PC1 axis yet at distinct values, suggesting that these represent different magnitudes of exploration/avoidance. The corridor variables (CORR1-CORN-CORR2, hereafter CORRS) were located at a distinct position on the PC2 axis, which appeared to be related to locomotory activity.

### Behavior of Female and Male AB and Wild Zebrafish in the zebrafish Multivariate Concentric Square Field

#### Locomotory Activity in the zebrafish Multivariate Concentric Square Field

**FIGURE 3:**
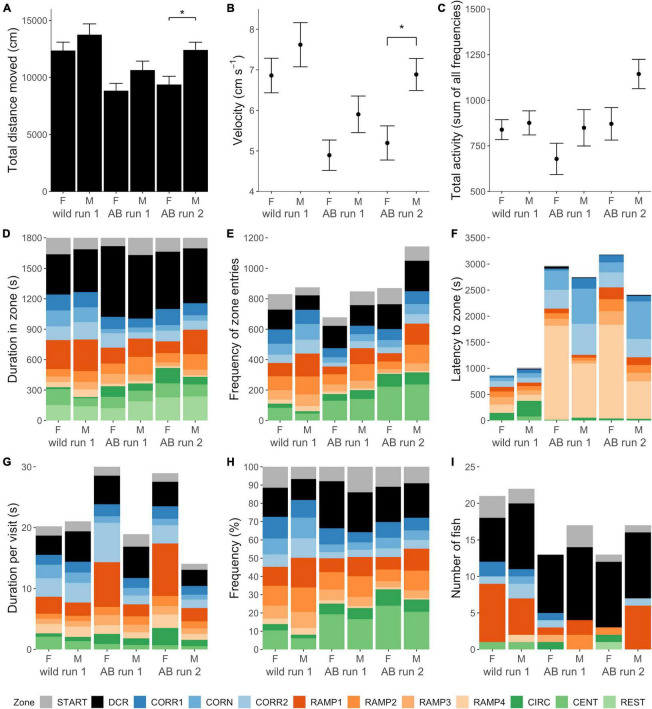
Behavior in the zMCSF test. Locomotory activity variables **(A)** total distance moved (cm), **(B)** average velocity (cm s^– 1^) and **(C)** total activity (sum of all zone entries) per experimental group calculated for the whole arena. In **(A–C)**, the height of the bars or points represents the mean ± SEM per group. Brackets with stars (*) indicate statistically significant differences (*p* < 0.05) between experimental groups; main effects of Strain, Sex, and Run are shown in Supplementary Table 1. **(D–I)** Present zone related variables **(D)** duration (s) in zone, **(E)** frequency of zone entries, **(F)** Latency (s) to zone, **(G)** average duration per visit (s), **(H)** percentage of zone entries, and **(I)** the number of fish that had a certain zone as its home base. For example, the majority of AB females in run 1 had the DCR as their home base, so the DCR was zone that the fish spent the longest cumulative duration in. In **(D–I)**, the height of each colored bar represents the mean per zone except for **(F)**, where these represent median latency (the more accurate summarizing statistic for this variable). For the REST zone, only duration could be quantified. Note that this figure combines the “strain” and “retested” datasets which were analyzed in two separate models per response variable, see methods section “Statistical Analyses.” CENT, center; CIRC, central circle; CORN, corner; CORR, corridor; DCR, dark corner roof; REST, the part of the arena not designated to any other zone; F, female; M, male.

Wild zebrafish moved longer distances than AB [LMM, *F*_(1, 69)_ = 13.263, *p* < 0.001; Table 1, Supplementary Tables 1, 2, and Figure 3A], at higher velocity [ANOVA, *F*_(1, 69)_ = 14.010, *p* < 0.001; Figure 3B] while the number of zone entries (total activity) was equal between the strains [Negative binomial GLM, χ^2^_(1, 69)_ = 1.326, *p* = 0.250; Figure 3C]. There was a tendency for a main effect of Sex on distance moved, with males of both strains moving marginally longer distances than females [LMM, *F*_(1, 69)_ = 3.226, *p* = 0.077; Table 1, Supplementary Table 1, and Figure 3A].

**TABLE 1:** Pairwise comparisons between strains and sexes in the zones of the zMCSF in AB and wild (W) zebrafish, separating males and females.

Sex	Zone	Duration	Duration	Frequency	Frequency	Latency
		(s)	per		(%)	(s)
			visit (s)			
Females	START					
	DCR					
	CORR1					
	CORN	W > AB***				W < AB*
	CORR2	W > AB*				
	RAMP1					
	RAMP2					
	RAMP3					
	RAMP4	W > AB***				W < AB**
	CIRC		W < AB*			
	CENT					
	REST					
Males	START				W < AB*	
	DCR					
	CORR1	W > AB*				
	CORN	W > AB***		W > AB**	W > AB***	W < AB**
	CORR2	W > AB***		W > AB**	W > AB**	
	RAMP1	W > AB*				
	RAMP2					
	RAMP3					
	RAMP4	W > AB***		W > AB***	W > AB**	W < AB*
	CIRC			W < AB**	W < AB***	
	CENT			W < AB*	W < AB***	
	REST					

Texts in cells indicate which of the experimental groups had a significantly higher or lower value of the measured zone-related variable. *p < 0.05, **p < 0.01, ***p < 0.001 comparing AB and wild within sex. CENT, center; CIRC, central circle; CORN, corner; CORR, corridor; DCR, dark corner roof; REST, the part of the arena not designated to any other zone.

The PCA was unable to separate the data from the repeated runs of testing for the AB strain zebrafish (Supplementary Figure 2A). Nevertheless, the loading plot showed a clear separation of the zones (Supplementary Figure 2B), highly similar to the PCA on the data from both strains (Figure 2B).

#### Explorative Behavior in the zebrafish Multivariate Concentric Square Field

There were significant differences between the zones of the zMCSF in duration, duration per visit, frequency, relative frequency and latency to visit (main effect of Zone, Supplementary Table 1). Wild zebrafish spent the longest duration in the DCR and shortest in the CIRC, in the order: DCR = RAMP1 = REST = CORN = CORR2 = CORR1 = START = RAMP2 = CENT = RAMP3 = RAMP4 > CIRC (Supplementary Table 2 and Figure 3D). AB zebrafish also spent the longest duration in the DCR, but the zone visited for the shortest duration was RAMP4, in the order DCR > RAMP1 = REST > RAMP2 = START = CENT = CORR1 = CORR2 = CIRC = RAMP3 = CORN > RAMP4 (Supplementary Table 2 and Figure 3D).

There was a significant main effect of Strain for response variables duration, frequency and latency, and a significant Zone by Strain interaction for all zone-related response variables (Supplementary Table 1), indicating that the strains allocated their time differently across the zMCSF zones. The pair-wise differences between the strain/sex groups are summarized in Table 1, Supplementary Table 2 and Figure 3A–I. Compared to AB, wild zebrafish spent more time in CORR1, CORN and CORR2 [LMM contrasts, *t*_(11, 622)_ = –3.220, *p* = 0.001; *t*_(11, 622)_ = –6.663, *p* < 0.001; *t*_(11, 622)_ = -5.348, *p* < 0.001; Figure 3D] and in RAMP1, 3, and 4 [*t*_(11, 622)_ = –3.238, *p* = 0.001; *t*_(11, 622)_ = –2.588, *p* = 0.010; *t*_(11, 622)_ = –6.654, *p* < 0.001; Figure 3D]. By contrast, wild zebrafish spent a shorter duration in CIRC [*t*_(11, 622)_ = –3.513, *p* = 0.001; Figure 3D] and a shorter average duration per visit in the DCR [*t*_11, 654)_ = –2.463, *p* = 0.001; Figure 3G]. The differences between the strains were always in the same direction for both sexes, but in some cases were only detected in one sex (Supplementary Table 2). A greater number of strain differences was detected in males compared to females (Table 1 and Supplementary Table 2). Notably, compared to AB males, wild males paid more visits to RAMP4 and fewer visits to CIRC and CENT, while this strain difference was absent in females (Table 1 and Supplementary Table 2).

Time series plots (Supplementary Figure 3) suggested that AB explored the arena during the first 10 min, spending more time in the RAMP zones early on, only to reside in the DCR for the remaining 20 min. Wild zebrafish divided their activity and time more equally over the different zones and spent less time in DCR, especially later in the test (Supplementary Figure 3).

**FIGURE 4:**
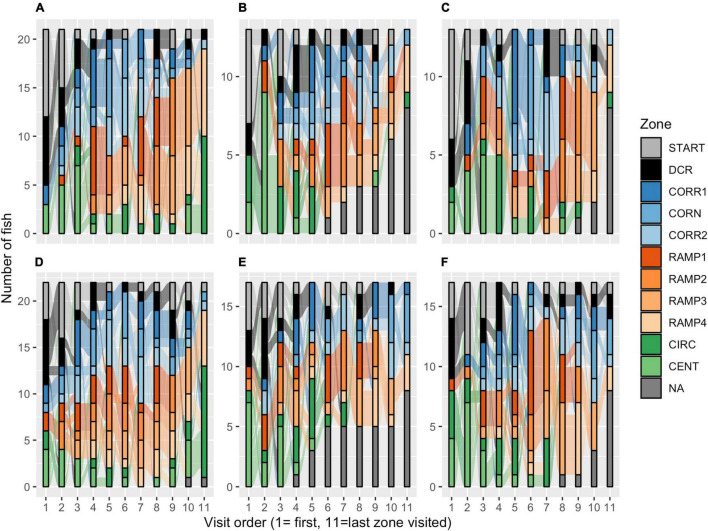
Sankey diagrams of explorative strategies in the zMCSF test. The diagrams represent the order in which the zones were visited for **(A)** wild females, **(B)** AB females run 1, **(C)** AB females run 2, **(D)** wild males, **(E)** AB males run 1, and **(F)** AB males run 2. The height of each colored bar represents the number of fish that visited a particular zone as the *n*th novel zone they visited. The flows between the bars describe the number of individuals that moved from one zone to the next novel zone. The gray bars (“NA”) represent the number of fish that did not have an *n*th zone, i.e., they did not explore more than *n*-1 zones. Example: in **(A)**, seven wild females were first detected in DCR; 5 of those fish then went on to the START zone while the other two moved to CORR1. CENT, center; CIRC, central circle; CORN, corner; CORR, corridor; DCR, dark corner roof; REST, the part of the arena not designated to any other zone; F, female; M, male.

AB zebrafish had a more pronounced preference for the DCR as a home base (53% of fish), while in wild zebrafish 35% of fish preferred the DCR and 30% the RAMP1 as a home base, a strain difference that was borderline significant [Poisson GLM, χ^2^_(1, 23)_ = 18.373, *p* = 0.073; Figure 3I]. No sex differences were detected in the number of zones visited within either strain [Poisson GLM contrast, AB: z_1_,_69_ = 0.368, *p* = 1.000; Wild: z_(1, 69)_ = 0.045, *p* = 1.000]. While AB zebrafish often left one or more zones unexplored (Figures 4B,E), wild zebrafish explored all zones [Binomial GLM, χ^2^_(1, 72)_ = 28.443, *p* < 0.001], the only exception being one wild male that never entered CIRC (Figures 4A,D).

#### Functional Areas of the zebrafish Multivariate Concentric Square Field

Correlations between zones revealed that there were six areas in the arena, consisting of zones that were positively intercorrelated in terms of the duration of time spent in these zones. For AB zebrafish these areas were START, DCR, CORRS, RAMP1-4 (hereafter RAMP), CENT-CIRC and REST (Supplementary Table 3A). More active animals (high velocity and total activity) spent more time in the CORRS, RAMP and CIRC and less time in the DCR. Individuals that spent more time in the DCR spent less time on the RAMP and more time in CORR1, CORN and REST zones. CORRS and CIRC, CENT and REST zones were positively correlated. However, REST was negatively correlated with RAMP, differentiating it from the CIRC-CENT area (Supplementary Table 3A).

The same six areas of intercorrelated zones could be differentiated in wild zebrafish, with minor modifications (Supplementary Table 3B). In wild zebrafish, duration in CORR2 was not correlated with CORR1 and CORN (*p* = 0.138 and *p* = 0.263, respectively). Moreover, only duration in RAMP1-2 but not in RAMP3-4 was negatively correlated with DCR (Supplementary Table 3B). In AB, the REST zone was negatively correlated with RAMP (Supplementary Table 3A), while in wild zebrafish the CENT and RAMP zones were negatively correlated (Supplementary Table 3B).

### Effect of Repeated Testing in the zebrafish Multivariate Concentric Square Field

There was a tendency for increased locomotory activity from Run 1 to 2, although this effect only reached statistical significance for total activity [GLMM, *F*_(1, 28)_ = 6.648, *p* = 0.011; Supplementary Table 4]. The sex difference in locomotory activity became more pronounced after taking into account both runs, with AB males traveling longer distances [LMM, *F*_(1, 28)_ = 7.452, *p* = 0.011] at higher speed [LMM, *F*_(1, 28)_ = 7.466, *p* = 0.011] and exhibiting higher total activity [GLMM, *F*_(1, 28)_ = 5.098, *p* = 0.024; Supplementary Table 4].

On the second testing occasion, AB zebrafish again spent the longest duration in the DCR and shortest in RAMP4, in the order: DCR > RAMP1 > REST = CENT = START = CORR1 > CORN = CORR2 > RAMP2 = CIRC > RAMP3 > RAMP4 (LMM, *F*_(11, 308)_ = 10.516, *p* < 0.001; Supplementary Table 2 and Figure 3D). For duration and frequency in zone, there was a main effect of Run [LMM, *F*_(1, 644)_ = 22.866, *p* < 0.001 and *F*_(1, 512)_ = 8.150, *p* = 0.006, respectively; Supplementary Table 4]. However, the lack of a Zone by Run interaction for all zone-related variables indicated that AB zebrafish did not allocate their time differently over zones on the second testing occasion (Supplementary Table 4). Only a single pair-wise comparison between the runs was statistically significant: AB males had a shorter average duration per visit to DCR in Run 2 compared to Run 1 [LMM contrast, *t*_(10, 505)_ = 3.215, *p* = 0.008; Supplementary Table 2].

The six areas of correlated zones outlined in section “Behavior of Female and Male AB and Wild Zebrafish in the zebrafish Multivariate Concentric Square Field” could also be distinguished in the second run, but some relationships between areas changed (Supplementary Table 3A). Upon repeated testing, activity was no longer related to duration in RAMP and CIRC zones. Animals that stayed longer in the DCR now spent less time in the CORN and CORR2 zones. Also, the negative correlation between the REST and RAMP zones was no longer present (Supplementary Table 3A).

Consistency repeatability was significant for distance moved and velocity (*r* = 0.432, p = 0.014 and *r* = 0.446, *p* = 0.013, respectively; Supplementary Table 5). RAMP1 had high repeatability both in terms of duration (*r* = 0.508, p = 0.038), frequency (*r* = 0.376, *p* = 0.041) and frequency (%) (*r* = 0.533, *p* = 0.002; Supplementary Table 5). Significant repeatabilities were also seen for the neighboring zones, average duration per visit to CORR2 (*r* = 0.556, *p* = 0.001) and frequency (%) of entering RAMP2 (*r* = 0.379, *p* = 0.039). Finally, the frequency of visits to the DCR was repeatable (*r* = 0.497, *p* = 0.005) and the repeatability of frequency to CIRC was approaching the significance level (*r* = 0.326, *p* = 0.079; Supplementary Table 5).

In the first run, the DCR was the home base for 60% of AB zebrafish (Figure 3I) and this preference was not altered by repeated testing [Poisson GLM, χ^2^_(1, 57)_ = 0.000, *p* = 1.000]. There was no significant effect of Run, Sex or their interaction on the number of zones explored [Poisson GLM, Run: χ^2^_(1, 58)_ = 2.251, *p* = 0.134; Sex: χ^2^_(1, 57)_ = 0.041, *p* = 0.840; Run by Sex: χ^2^_(1, 56)_ = 0.100, *p* = 0.752], nor whether or not all zones had been entered [Binomial GLM, Run: χ^2^_(1, 58)_ = 0.000, *p* = 1.000; Sex: χ^2^_(1, 58)_ = 1.248, *p* = 0.264; Run by Sex: χ^2^_(1, 58)_ = 0.000, *p* = 1.000]. In both runs, 8 AB females and 8 AB males (53%) left one or more zones unexplored (Figure 4).

### Behavior of Female and Male AB and Wild Zebrafish in the Novel Tank Diving Test

**FIGURE 5:**
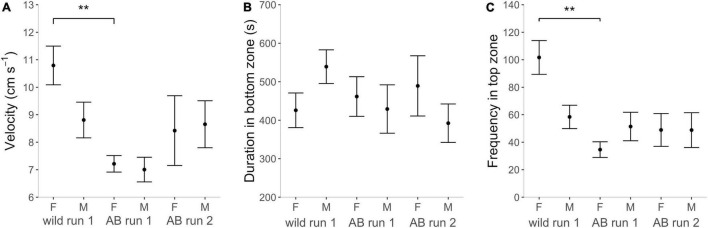
Behavior in the novel tank diving test (NTDT). **(A)** Velocity (cm s^– 1^) in the whole arena, **(B)** duration (s) in the bottom third of the arena for the experimental groups, and **(C)** frequency of entries into the top third (from left to right in each graph) wild females, wild males, AB females run 1, AB males run 1, AB females run 2 and AB males run 2. Note that this figure combines the “strain” and “retested” datasets which were analyzed in two separate models per response variable, see methods section “Statistical Analyses.” Brackets with stars indicate significance with **p < 0.01.

In the NTDT, there was a main effect of Strain on all activity variables and a main effect of Sex on distance moved and velocity (Supplementary Table 1). Wild zebrafish had higher locomotory activity than AB as indicated by a longer distance moved [ANOVA, *F*_(1, 69)_ = 53.091, *p* < 0.001; Table 2 and Supplementary Table 6], higher velocity [ANOVA, *F*_(1, 69)_ = 18.563, *p* < 0.001; Figure 5A] and higher total activity [GLM, χ^2^_(1, 71)_ = 11.422, *p* < 0.001; Supplementary Table 6]. In both strains, females moved longer distances [ANOVA, *F*_(1, 69)_ = 9.501, *p* < 0.003) and at higher speed than males [ANOVA, *F*_(1, 69)_ = 4.214, *p* = 0.044], although there was no sex difference in total activity [GLM, χ^2^_(1, 70)_ = 0.446, *p* = 0.504; Supplementary Table 6].

**TABLE 2:** Pairwise comparisons between strains and sexes in the zones of the novel tank diving test (NTDT) in AB and wild (W) zebrafish, separating males and females.

Sex	Zone	Duration (s)	Duration per visit (s)	Frequency	Frequency (%)	Latency (s)
Females	TOP			W > AB***		
	MID			W > AB***		
	BOT					
Males	TOP					
	MID					
	BOT					

Texts in cells indicate which of the experimental groups had a significantly higher or lower value of the measured zone-related variable. ***p < 0.001.

Both zebrafish strains spent most time in the bottom and least time in the top third of the NTDT [LMM contrast, AB TOP vs. BOT, *t*_(2, 179)_ = –4.053, *p* = 0.001; wild Top vs. Bottom, *t*_(2, 179)_ = –7.066, *p* < 0.001; Supplementary Table 6 and Figure 5B]. There was a main effect of Strain and a Zone by Strain interaction for some zone-related variables (Supplementary Table 1), indicating that there were some differences in how much time each strain spent in the top, middle and bottom third of the arena. *Post-hoc* tests showed that wild females entered the top and middle third more often than AB females [GLMM contrast, *z*_(1, 205)_ = –4.830, *p* < 0.001; *z*_(1, 205)_ = –3.805, *p* < 0.001], while wild and AB males did not differ [GLMM contrast, *z*_(1, 205)_ = –0.628, *p* = 1.000; *z*_(1, 205)_ = –2.005, *p* = 0.270; Table 2 and Figure 5C].

Repetition of the NTDT for AB fish one week later revealed a tendency for an increase in velocity [LMM, *F*_(1, 28)_ = 3.791, *p* = 0.061] and total activity from Run 1 to 2 [GLMM, *F*_(1, 28)_ = 3.311, *p* = 0.096, Supplementary Tables 4, 6]. We did not detect a main effect of Run nor a Zone by Run interaction for any of the zone-related response variables (Supplementary Table 4).

### Comparison of Behavior of AB Zebrafish in zebrafish Multivariate Concentric Square Field and Novel Tank Diving Test

In AB, correlations between duration spent in each zone of the zMCSF and NTDT revealed that in Run 2, duration in bottom of the NTDT correlated negatively with duration in RAMP2, 3, and 4 and duration in the top correlated positively with RAMP 2 and 3 (Supplementary Table 3A). Furthermore, duration in bottom correlated positively with DCR (Supplementary Table 3A).

## Discussion

Behavioral tests continue to play an important role for the discovery of novel neuroactive compounds and in the development of zebrafish models of neurological and neuropsychiatric disease. Classical tests, such as the open field, shelter, light/dark, elevated plus maze and NTDT, are still widely used since they enable comparisons with previous studies and generally translate well to other study species (in particular rodents). To ensure a broad behavioral screening, many studies make use of a sequence of classical tests (a “test battery”), but evidence is accumulating that animals habituate to the novelty offered in the same test (Rodgers, 1997) and even generalize experiences between tests (McIlwain et al., 2001; Blokland et al., 2012). Herein, we have developed a complex testing environment for the quantification of explorative behavior in adult zebrafish, the zebrafish Multivariate Concentric Square Field (zMCSF), that combines elements of classical tests in a single arena and greatly reduces the number of tests performed.

We previously optimized the dimensions of the zMCSF arena (Roman et al., 2016, 2018; Bikovski et al., 2020). Here, we further optimized the division of the arena into zones as compared to the previous version (Roman et al., 2018) by minimizing the area designated as the REST zone, by dividing the central square into the central circle (CIRC) and the surrounding area (CENT) and by dividing the inclined ramp into four rather than two zones. Although a formal comparison between the zone division settings is outside of the scope of the current study, we would like to emphasize that the current zone division further refined the study of explorative strategies and their behavioral interpretation as compared to previous versions (Roman et al., 2018). The division of the central square into an outer and central zone provided a closer comparison to the classical open field test, and the subdivision of the ramp enabled a more detailed study of risk-taking behavior.

### Behavior of AB and Wild Zebrafish in the zebrafish Multivariate Concentric Square Field

We detected considerable differences in explorative behavior between laboratory and wild zebrafish. Notably, wild zebrafish swam faster and explored all zones, while AB zebrafish often left one or more zones unexplored. Wild zebrafish showed stronger avoidance of the open area in the center, but entered the shallowest zone in the arena, RAMP4, earlier. The strains did not differ in the time spent in the sheltered area (DCR). In nature, open areas are associated with increased predation risk for individual prey fish (Ruxton and Johnsen, 2016) and wild zebrafish occur in small vegetated streams feeding on insects, moving into shallow flooded areas for spawning at the start of the rainy season (Engeszer et al., 2007; Sundin et al., 2019). By contrast, the environment of laboratory zebrafish has for many generations consisted of deep tanks with no shallow areas and no vegetation cover (Sessa et al., 2008). It is possible that laboratory zebrafish over generations have become less selective with regards to the exact housing conditions, while in nature selection pressures for avoiding open water and exploring shallow water continued to act. This could explain the greater avoidance of the center zone and greater exploration of the shallow zones of the ramp by the wild strain. Indeed, the strain differences detected in the zMCSF imply that the behavioral adaption to the laboratory environment is characterized by relaxed aversion to risky areas with no potential gain (open area) and reduced exploration of risky areas with potential gain (the inclined ramp), while attraction to safe areas seems to be unaltered. These findings are congruent with the idea that domesticated animals are characterized by a low propensity to perform active behaviors (Van Reenen et al., 2005), and low stress reactivity, in other words, are “docile” rather than “shy” (Koolhaas et al., 2010; Koolhaas and Van Reenen, 2016; Rauw et al., 2017).”

The results from the zMCSF correspond well to the behavior of zebrafish in classical tests. The lower velocity and avoidance of the center of an open field by domesticated zebrafish has been reported before (Baker et al., 2018) and extends to other anti-predator behaviors, including reduced diving responses and altered responses to conspecific alarm pheromone (Mustafa et al., 2019; Vossen et al., 2020b). Moreover, the absence of large strain differences in DCR duration is in line with the absence of a strain difference in the shelter test in the same population of wild zebrafish (Mustafa et al., 2019). Thus, the strain differences we observed in the zMCSF appear to be comparable to those reported using single tests. However, the zMCSF adds the advantage of reducing the need for repeated testing, which makes the zMCSF more time-efficient, reduces handling stress and minimizes possible carry-over effects.

Comparing these results to wild and inbred lines of laboratory mice, it becomes apparent that the strong avoidance of the central zone in wild animals is a particular consistent finding across species (Augustsson and Meyerson, 2004; Augustsson et al., 2005). A higher duration and frequency of visits to the slope (corresponding to RAMP1) has also been observed in wild mice in comparison to the BALB/c laboratory line, although no differences were observed with regard to the bridge (Augustsson and Meyerson, 2004; Augustsson et al., 2005). Similar to zebrafish, the duration in DCR was not different across wild and laboratory lines of mice (Augustsson and Meyerson, 2004; Augustsson et al., 2005). Hence, actively seeking shelter does not appear to be a major factor differentiating wild and laboratory bred animals, at least not in the current test environment in which animals can freely choose where to reside. It should be noted, however, that some wild zebrafish may have considered the RAMP1 zone an (additional) home base, in which case wild zebrafish may have spent more time in the safety of their home base than AB zebrafish. We found only one discrepancy in MCSF behavior between the species; wild zebrafish showed an increased duration in the corridors, while wild mice showed a lower use of this area compared to the laboratory lines. The corridors present a semi-sheltered area where movement is possible (Roman and Colombo, 2009), therefore we suggest that this difference is driven by the higher velocity of wild zebrafish. Wild mice did not have a higher velocity than the laboratory lines, explaining the absence of a difference in the corridor duration for this species. Mice and zebrafish may indeed differ at the species level in the propensity to respond reactively (freeze) or proactively (swimming) when entering a novel environment.

#### Sex Differences in the zebrafish Multivariate Concentric Square Field

We found no major differences between females and males of each strain. Locomotory activity was similar in the sexes in run 1. Studies using classical tests often report that AB males had a higher velocity than females (Vossen et al., 2016, Vossen et al., 2020b; Mustafa et al., 2019). In the zMCSF the sex difference in velocity was only marginally significant in the first run. This may be explained by the larger size and/or presence of physical structure in the zMCSF, which limits repetitive movement along the walls. Indeed, upon inclusion of the second run of the zMCSF, velocity of AB males was higher than AB females (see section “Effect of Repeated Testing in the zebrafish Multivariate Concentric Square Field”), suggesting that the effect on locomotion is smaller in the zMCSF but can be still detected for larger samples.

The sexes did not show any major difference in the duration of time spent in and number of visits to any of the zMCSF zones. Previous studies have reported sex effects on risk-taking behaviors in different directions. Wild females exhibited increased shelter seeking and thigmotaxis (Dahlbom et al., 2011), and a stronger diving response to conspecific alarm pheromone (Vossen et al., 2020b), while another study on wild zebrafish reported no sex differences in shelter seeking and bottom dwelling (Mustafa et al., 2019). A recent study reported an opposite effect of a pharmaceutical (scopolamine) on anxiety-like behavior in males and females of the short-fin strain (Dos Santos et al., 2021). Although studies of “wild-caught” zebrafish are complicated by the different origin of the fish and should therefore be interpreted with caution, there does seem to be a disparity between studies in the direction and magnitude of sex differences reported. This may be because sex differences are few and/or of small effect. Alternatively, the differences between the zMCSF and classical tests may reflect the design of the test arena. Their higher activity may “drive” males into the center of the open field, the top zone of the NTDT, or the white compartment of the light/dark test, simply because there are no other zones to move in. In the zMCSF, the shelter and the risky areas only comprise a small part of the arena, therefore a move into this area may be interpreted as a more active choice for exploration. Hence the zMCSF may allow for a clearer separation between locomotion and explorative behavior.

Finally, it is worth mentioning that some of the strain differences were more pronounced in males than in females. Compared to AB males, wild males paid more visits to the corridors and RAMP4, and less visits to the central area, whilst these effects did not reach statistical significance for the comparison between AB and wild females. This may imply that (the response to) selection for the domesticated environment is stronger in zebrafish males compared to females.

### Effect of Repeated Testing in the zebrafish Multivariate Concentric Square Field

Overall, we found only minor changes in explorative behavior when the AB zebrafish were tested in the zMCSF 1 week later, and the few differences we found were confined to males. AB males were more active than females in the second run, an effect that has also been reported for the open field using the same interest interval (Thomson et al., 2020). In rodents, activity during the second test occasion is dependent on developmental stage. Adult rats were less active in the MCSF upon retesting (Meyerson et al., 2006; Momeni and Roman, 2014), while adolescent rats increased the total number of visits to zones (Lundberg et al., 2019).

Male zebrafish decreased their visit duration to the DCR, an effect that has been reported in rats by some studies (Momeni and Roman, 2014; Lundberg et al., 2019), whereas others found no effect (Meyerson et al., 2006; Roman and Colombo, 2009). A consistent finding in rats is a decrease in duration and/or frequency of visits to the hurdle, slope and bridge in the second trial (Meyerson et al., 2006; Roman and Colombo, 2009; Momeni and Roman, 2014; Lundberg et al., 2019), and in most of these studies, an increase in visits to the corridors. We did not detect these same effects in zebrafish, which may reflect underlying differences in episodic/spatial memory between the zebrafish and rodents. Zebrafish can be taught to associate a color with a mild electric shock within a single training session but fail to show avoidance of this color when tested again 1 week later (Vossen et al., 2020). By contrast, rats can spatially locate exposure to an aversive stimulus when repeatedly tested 2 weeks later (Karlsson et al., 2009).

Significant repeatabilities of *r*∼0.5 were found for activity variables as well as for the DCR and RAMP1 zones, which is considered to be a high repeatability (Bell et al., 2009). High consistency of velocity has also been reported for zebrafish repeatedly tested in the open field (Toms and Echevarria, 2014; Baker et al., 2018; Fangmeier et al., 2018; Thomson et al., 2020). Regarding responses to novelty, high consistency of inspection duration has also been reported for zebrafish repeatedly tested in the mirror (Toms and Echevarria, 2014), novel object and predator tests (Toms and Echevarria, 2014; Fangmeier et al., 2018). The high consistency of RAMP1 is interesting since rodents frequently display stretched attend posture in this zone (the slope in the rodent MCSF), which is a key part of risk assessment behavior (Macintosh and Grant, 1963; Rodgers et al., 1999; Augustsson and Meyerson, 2004). Under the assumption that the RAMP1 (zMCSF) and the slope (rodent MCSF) are homologous, this would imply that zebrafish show equal risk assessment in repeated testing while risk taking differs.

### Comparison to the Novel Tank Diving Test

In line with the usual pattern reported for the NTDT (Levin et al., 2007), both AB and wild zebrafish avoided the top zone. However, wild zebrafish paid more visits to the middle and top third of the tank compared to AB zebrafish. High levels of bottom dwelling in the AB strain have been reported before (Gerlai et al., 2008), although it is not clear whether this accurately reflects increased anxiety-like behavior of this strain (Vossen et al., 2020b). The positive correlation between duration in bottom (NTDT) and DCR (zMCSF) suggests that these zones contain elements of safety. The positive correlation between the number of visits to the TOP (NTDT) and RAMP (zMCSF) was expected, since both zones are located close to the water surface, an area associated with a risk for avian predation (Levin et al., 2007).

At a first glance, the overall conclusions from the zMCSF and NTDT seem to agree that the strongest differences were those between the strains and no or minor effects were seen between the sexes and between repetitions of the zMCSF. The differences between the strains were furthermore in the same direction in both tests, with wild zebrafish exhibiting higher activity than AB and moving more in shallow water. However, upon a closer look it becomes apparent that a much smaller number of effects was detected in the NTDT, even after correcting for the lower number of zones in this arena. Apart from the strain difference in activity, the NTDT revealed only two differences (wild females paid more visits to the middle and top), whereas in the zMCSF 21 strain differences were detected (17 in males and 4 in females), related to 7 zones. Indeed, this suggests that the zMCSF, like the rodent MCSF, constitutes a more nuanced behavioral test.

Yet the distinction between the zMCSF and NTDT goes deeper than just a quantitative difference in the number of effects detected. The zMCSF was able to distinguish between two independent risk-related behaviors, which are intertwined in the NTDT. Paradoxically, in the zMCSF the wild strain displayed more elaborate exploration and entered the shallowest zone more readily, while it simultaneously avoided the open areas more than AB zebrafish. In the NTDT, only a reduced diving response of wild zebrafish was detected, which (when viewed in isolation) could lead to the erroneous conclusion that wild zebrafish “showed reduced anxiety-like behavior” or “were more bold/risk taking,” which is only one side of the coin. Taking into account both tests, it rather seems that while wild zebrafish are more active explorers that avoid shallow areas less, they also display more elaborate risk assessment and are more sensitive to certain kinds of risk (i.e., open areas). Indeed, the results from the zMCSF suggest that risk taking should be considered in relation to the nature of the challenge.

### Interpretation of the zebrafish Multivariate Concentric Square Field Areas in Terms of Risk and Safety

Our findings indicate that the interpretation of the MCSF areas in terms of risk and safety is largely conserved between rodents and zebrafish. The DCR may be considered the safest zone of the zMCSF, since it often was the zone that was visited first, with the longest cumulative duration, indicative of a home base (Eilam and Golani, 1989; Stewart et al., 2010). The NTDT provided cross-validation for this interpretation since the duration in the DCR was positively correlated with the duration in the bottom zone. We suggest that the corridors (CORR1, CORN, CORR2) also contains aspects of safety, but in contrast to the DCR the fish can swim larger distances in this relatively sheltered area. In the corridors motor restlessness can be expressed without affecting measures of active exploration, which has also been reported for rats (Roman and Colombo, 2009). The RAMP is an area of gradually decreasing water depth, zebrafish spent little time here and were most hesitant to enter this area (especially the shallowest part, RAMP4), AB more so than wild zebrafish. The RAMP has an inverse relationship to the DCR especially in AB zebrafish, providing further evidence for an interpretation as a high-risk zone. Also the center of the arena (CIRC and CENT) may be a high-risk zone, which in particular wild zebrafish are hesitant to enter and spend little time in, while AB zebrafish more readily move through it much like movement in the corridors, suggesting the interpretation of this area is more strain dependent. Hence the RAMP and arena center may each reflect different “qualities” in terms of risk assessment and risk taking (Roman et al., 2012; Meyerson et al., 2013). Further experiments using pharmacological pre-treatments with for instance anxiolytic substances such as benzodiazepines (Bencan et al., 2009) are needed to further validate the here proposed interpretation of the zMCSF zones.

## Conclusion

The zMCSF constitutes a multifaceted test environment to quantify zebrafish explorative behavior and behavioral profiles, containing zones associated with different kinds and magnitudes of risk and safety. We here report that exploratory behavior in the zMCSF was qualitatively different between laboratory and wild zebrafish, while sex differences within strains were small and most pronounced in the AB strain. Our results suggest that the zMCSF is a more precise behavioral tool, able to detect small differences between the zebrafish strains and sexes that were not picked up in the more reductionistic NTDT. Simultaneously, the zMCSF provides a wider, more comprehensive perspective on exploration and risk-taking. An added benefit is the apparent high repeatability and low habituation to the zMCSF, as assessed from the single repetition described here. Additional pharmacological validation and cross-validation of the zMCSF with other classical tests (e.g., open field, shelter test, plus maze) may further substantiate the interpretation of behavior in the zMCSF.

## Data Availability Statement

The datasets generated for this study can be found on figshare with the identifier https://doi.org/10.6084/m9.figshare.15022482.

## Ethics Statement

The animal study was reviewed and approved by the Uppsala Regional Animal Ethical Committee (permit C55/13), following the guidelines of the Swedish Legislation on Animal Experimentation (Animal Welfare Act SFS1998:56) and the European Union Directive on the Protection of Animals Used for Scientific Purposes (Directive 2010/63/EU).

## Author Contributions

ER conceptualized, designed the study, and supervised RB and PR. SW provided supervision and resources. RB and PR performed the experiments. LV validated, curated, visualized the data, conducted the statistical analyses, and wrote the first draft of the manuscript. All authors contributed to manuscript revision, read, and approved the submitted version.

## Conflict of Interest

The authors declare that the research was conducted in the absence of any commercial or financial relationships that could be construed as a potential conflict of interest.

## Publisher’s Note

All claims expressed in this article are solely those of the authors and do not necessarily represent those of their affiliated organizations, or those of the publisher, the editors and the reviewers. Any product that may be evaluated in this article, or claim that may be made by its manufacturer, is not guaranteed or endorsed by the publisher.

## Abbreviations

CENT: Center zone of the zMCSF
CIRC: Central circle zone of the zMCSF
CORN: Corner zone of the zMCSF
CORR1: Corridor 1 zone of the zMCSF
CORR2: Corridor 2 zone of the zMCSF
CORRS: Corridor 1, corner and corridor 2 of the zMCSF
DCR: Dark corner roof zone of the zMCSF
MCSF: Multivariate Concentric Square Field ™
NTDT: Novel tank Diving Test
PCA: Principal Component Analysis
RAMP: Inclined ramp of the zMCSF
REST: The part of the zMCSF not designated to any other zone
START: Start zone of the zMCSF
zMCSF: zebrafish Multivariate Concentric Square Field ™.
