# Supplementary material for: The zebrafish Multivariate Concentric Square Field: A Standardized Test for Behavioral Profiling of Zebrafish (Danio rerio)
Source: Front Behav Neurosci. 2022 Mar 17;16:744533. doi: 10.3389/fnbeh.2022.744533 (PMC8968638; doi:10.3389/fnbeh.2022.744533)
Supplement: Supplementary file 1 [file Data_Sheet_1.docx]

Supplementary Material

# Supplementary Tables

**Supplementary Table 1.** Results from the models on the effect of Zone, Strain, Sex and their interactions on each response variable in both the zMCSF and novel tank diving test (NTDT). F-values, nominator and denominator degrees of freedom (in subscript) and p-values are presented.

| Response | Explanatory | zMCSF | | NTDT | |  |
| --- | --- | --- | --- | --- | --- | --- |
| Distance moved | Strain | F_1,69_=13.263 | p<0.001 | F_1,69_=50.528 | p<0.001 | |
| (cm) | Sex | F_1,69_=3.226 | p=0.077 | F_1,69_=9.501 | p=0.003 | |
|  | Strain × Sex | F_1,69_=0.056 | p=0.811 | F_1,69_=0.008 | p=0.930 | |
| Velocity (cm s^-1^) | Strain | F_1,69_=14.010 | p<0.001 | F_1,69_=17.562 | p<0.001 | |
|  | Sex | F_1,69_=2.505 | p=0.118 | F_1,69_=4.214 | p=0.044 | |
|  | Strain × Sex | F_1,69_=0.065 | p=0.800 | F_1,69_=2.011 | p=0.161 | |
| Total activity | Strain | χ^2^_1,69_=1.326 | P=0.250 | χ^2^_1,69_=11.422 | p<0.001 | |
|  | Sex | χ^2^_1,69_=1.457 | p=0.227 | χ^2^_1,69_=0.446 | p=0.504 | |
|  | Strain × Sex | χ^2^_1,69_=0.852 | p=0.356 | χ^2^_1,69_=5.978 | p=0.014 | |
| Duration (s) | Zone | F_11,759_=31.065 | p<0.001 | F_2,207_=29.096 | p<0.001 | |
|  | Strain | F_1,69_=22.864 | p<0.001 | F_1,207_=2.269 | p=0.134 | |
|  | Sex | F_1,69_=0.096 | p=0.757 | F_1,207_=2.280 | p=0.133 | |
|  | Zone × Strain | F_11,759_=10.638 | p<0.001 | F_2,207_=1.040 | p=0.355 | |
|  | Zone × Sex | F_11,759_=0.775 | p=0.666 | F_2,207_=0.218 | p=0.804 | |
|  | Strain × Sex | F_1,69_=0.056 | p=0.814 | F_1,207_=0.006 | p=0.941 | |
|  | Zone × Strain × Sex | F_11,759_=0.517 | p=0.893 | F_2,207_=2.406 | p=0.093 | |
| Duration per | Zone | F_10,636_=38.178 | p<0.001 | F_2,179_=51.392 | p<0.001 | |
| visit (s) | Strain | F_1,69_=0.725 | p=0.398 | F_1,1_=2.640 | p=0.404 | |
|  | Sex | F_1,69_=2.552 | p=0.115 | F_1,68_=0.747 | p=0.391 | |
|  | Zone × Strain | F_10,636_=4.978 | p<0.001 | F_2,179_=7.926 | p=0.001 | |
|  | Zone × Sex | F_10,636_=2.212 | p=0.016 | F_2,179_=0.212 | p=0.809 | |
|  | Strain × Sex | F_1,69_=2.919 | p=0.092 | F_1,68_=6.943 | p=0.010 | |
|  | Zone × Strain × Sex | F_10,636_=0.195 | p=0.195 | F_2,179_=2.564 | p=0.079 | |
| Frequency | Zone | F_10,757_=27.599 | p<0.001 | F_2,205_=21.309 | p<0.001 | |
|  | Strain | F_1,69_=5.860 | p=0.035 | F_1,205_=23.000 | p<0.001 | |
|  | Sex | F_1,69_=0.185 | p=0.594 | F_1,205_=0.687 | p=0.409 | |
|  | Zone × Strain | F_10,757_=12.036 | p<0.001 | F_2,205_=4.238 | p=0.019 | |
|  | Zone × Sex | F_10,757_=1.435 | p=0.077 | F_2,205_=1.058 | p=0.355 | |
|  | Strain × Sex | F_1,69_=0.283 | p=0.557 | F_1,205_=12.462 | p<0.001 | |
|  | Zone × Strain × Sex | F_10,757_=1.757 | p=0.120 | F_2,205_=0.941 | p=0.390 | |
| Frequency (%) | Zone | F_10,757_=28.028 | p<0.001 | F_2,205_=70.053 | p<0.001 | |
|  | Strain | F_1,69_=2.938 | p=0.094 | F_1,205_=0.016 | p=0.883 | |
|  | Sex | F_1,69_=0.000 | p=0.815 | F_1,205_=0.002 | p=0.977 | |
|  | Zone × Strain | F_10,757_=10.639 | p<0.001 | F_2,205_=8.564 | p<0.001 | |
|  | Zone × Sex | F_10,757_=2.065 | p=0.023 | F_2,205_=3.518 | p=0.030 | |
|  | Strain × Sex | F_1,69_=0.160 | p=0.689 | F_1,205_=0.004 | p=0.951 | |
|  | Zone × Strain × Sex | F_10,757_=1.665 | p=0.083 | F_2,205_=1.271 | p=0.280 | |
| Latency (s) | Zone | F_10,690_=24.185 | p<0.001 | F_2,207_=26.507 | p<0.001 | |
|  | Strain | F_1,69_=11.018 | p=0.001 | F_1,207_=4.075 | p=0.045 | |
|  | Sex | F_10,690_=1.046 | p=0.310 | F_1,207_=0.301 | p=0.584 | |
|  | Zone × Strain | F_10,690_=6.199 | p<0.001 | F_2,207_=2.165 | p=0.117 | |
|  | Zone × Sex | F_10,690_=1.568 | p=0.112 | F_2,207_=0.187 | p=0.830 | |
|  | Strain × Sex | F_1,69_=0.349 | p=0.557 | F_1,207_=0.382 | p=0.537 | |
|  | Zone × Strain × Sex | F_10,690_=0.836 | p=0.836 | F_2,207_=0.638 | p=0.529 | |

**Supplementary Table 2.** Descriptive statistics per experimental group for the zMCSF test. Note that this table combines the “strain” and “retested” datasets which were analyzed in two separate models per response variable, see methods section 2.3.

|  |  | **Wild** | | | **AB Run 1** | | | **AB Run 2** | | |
| --- | --- | --- | --- | --- | --- | --- | --- | --- | --- | --- |
| **Zone** | **Variable** | **Females** | **Males** | **Females** | | **Males** | **Females** | | **Males** |  |
| ARENA | Sample size | 21 | 22 | 13 | | 17 | 13 | | 17 |  |
|  | Distance moved (cm) | 12325 ± 771 | 13713 ± 980 | 8804 ± 675 | | 10616 ± 812 | 9346 ± 757 | | 12379 ± 711**^#^** |  |
|  | Velocity (cm s^-1^) | 6.9 ± 0.4 | 7.6 ± 0.5 | 4.9 ± 0.4 | | 5.9 ± 0.5 | 5.2 ± 0.4 | | 6.9 ± 0.4**^#^** |  |
|  | Total activity | 839 ± 55 | 876 ± 66 | 679 ± 86 | | 849 ± 100 | 871 ± 89 | | 1144 ± 80 |  |
| START | Duration (s) | 157 ± 36 | 111 ± 27 | 81 ± 23 | | 167 ± 53 | 136 ± 38 | | 102 ± 24 |  |
|  | Duration per visit (s) | 2 ± 0 | 2 ± 0 | 1 ± 0 | | 2 ± 1^##^ | 1 ± 0 | | 1 ± 0 |  |
|  | Frequency | 96 ± 18 | 53 ± 8 | 56 ± 13 | | 90 ± 19 | 106 ± 27 | | 94 ± 12 |  |
|  | Frequency (%) | 11 ± 2 | 7 ± 1* | 8 ± 1 | | 14 ± 3 | 11 ± 2 | | 9 ± 1 |  |
|  | Latency (s) | 1 (0 – 25) | 23 (3 – 118) | 14 (0 – 91) | | 6 (1 – 18) | 0 (0 – 17) | | 5 (2 – 42) |  |
| DCR | Duration (s) | 411 ± 68 | 423 ± 58 | 698 ± 141 | | 628 ± 119 | 564 ± 97 | | 541 ± 90 |  |
|  | Duration per visit (s) | 3 ± 0 | 5 ± 1**^#^** | 4 ± 1 | | 5 ± 1 | 4 ± 0 | | 3 ± 0** |  |
|  | Frequency | 134 ± 15 | 96 ± 10 | 147 ± 27 | | 136 ± 22 | 164 ± 31 | | 200 ± 29 |  |
|  | Frequency (%) | 16 ± 2 | 12 ± 1 | 26 ± 5 | | 22 ± 4 | 19 ± 4 | | 19 ± 3 |  |
|  | Latency (s) | 4 (0 – 27) | 13 (0 – 107) | 44 (9 – 392) | | 11 (1 – 571) | 8 (2 – 278) | | 17 (0 – 94) |  |
| CORR1 | Duration (s) | 158 ± 29 | 150 ± 33* | 118 ± 38 | | 87 ± 27 | 158 ± 33 | | 119 ± 19 |  |
|  | Duration per visit (s) | 2 ± 0 | 2 ± 0 | 2 ± 0 | | 1 ± 0 | 2 ± 0 | | 1 ± 0 |  |
|  | Frequency | 99 ± 15 | 91 ± 23 | 60 ± 19 | | 54 ± 18 | 72 ± 14 | | 84 ± 15 |  |
|  | Frequency (%) | 12 ± 2 | 10 ± 2 | 9 ± 2 | | 5 ± 1 | 9 ± 1 | | 7 ± 1 |  |
|  | Latency (s) | 33 (16 – 64) | 71 (34 – 180) | 35 (23 – 390) | | 205 (61 – 763) | 141 (22 – 196) | | 106 (14 – 492) |  |
| CORN | Duration (s) | 157 ± 22 | 145 ± 18*** | 42 ± 18 | | 47 ± 18 | 54 ± 17 | | 52 ± 13 |  |
|  | Duration per visit (s) | 2 ± 0 | 2 ± 0 | 1 ± 0 | | 1 ± 0 | 1 ± 0 | | 1 ± 0 |  |
|  | Frequency | 76 ± 11 | 104 ± 18** | 34 ± 13 | | 47 ± 20 | 48 ± 12 | | 68 ± 16 |  |
|  | Frequency (%) | 9 ± 1 | 11 ± 1** | 4 ± 1 | | 4 ± 1 | 6 ± 1 | | 5 ± 1 |  |
|  | Latency (s) | 50 (15 – 122) | 85 (16 – 204) | 371 (32 – 1169) | | 674 (207 – 1800) | 192 (24 – 609) | | 721 (98 – 1383) |  |
| CORR2 | Duration (s) | 137 ± 37 | 173 ± 34*** | 143 ± 77 | | 62 ± 22 | 107 ± 31 | | 89 ± 21 |  |
|  | Duration per visit (s) | 3 ± 1 | 3 ± 1 | 5 ± 3 | | 1 ± 0**^##^** | 3 ± 1 | | 1 ± 0 |  |
|  | Frequency | 58 ± 8 | 92 ± 11** | 26 ± 9 | | 46 ± 18 | 38 ± 10 | | 62 ± 14 |  |
|  | Frequency (%) | 7 ± 1 | 11 ± 1** | 3 ± 1 | | 4 ± 1 | 5 ± 1 | | 5 ± 1 |  |
|  | Latency (s) | 81 (16 – 147) | 97 (35 – 218) | 360 (38 – 1169) | | 596 (137 – 1800) | 286 (131 – 890) | | 352 (36 – 888) |  |
| RAMP1 | Duration (s) | 280 ± 55 | 311 ± 47* | 157 ± 36 | | 182 ± 46 | 116 ± 30 | | 242 ± 47 |  |
|  | Duration per visit (s) | 3 ± 0 | 2 ± 0 | 7 ± 5 | | 2 ± 0 | 9 ± 7 | | 2 ± 1 |  |
|  | Frequency | 89 ± 11 | 150 ± 21 | 52 ± 11 | | 104 ± 29 | 55 ± 12 | | 139 ± 27 |  |
|  | Frequency (%) | 11 ± 1 | 16 ± 1 | 8 ± 2 | | 11 ± 3 | 7 ± 2 | | 12 ± 2 |  |
|  | Latency (s) | 72 (3 – 125) | 68 (5 – 199) | 88 (18 – 241) | | 53 (2 – 721) | 228 (6 – 356) | | 148 (6 – 262) |  |
| RAMP2 | Duration (s) | 71 ± 11 | 107 ± 22 | 101 ± 34 | | 171 ± 67 | 92 ± 31 | | 151 ± 34 |  |
|  | Duration per visit (s) | 1 ± 0 | 1 ± 0 | 1 ± 0 | | 1 ± 0 | 2 ± 0 | | 1 ± 0 |  |
|  | Frequency | 90 ± 13 | 118 ± 18 | 66 ± 21 | | 110 ± 39 | 50 ± 14 | | 122 ± 26 |  |
|  | Frequency (%) | 11 ± 2 | 13 ± 2 | 9 ± 3 | | 11 ± 4 | 6 ± 2 | | 11 ± 2 |  |
|  | Latency (s) | 89 (10 – 220) | 81 (13 – 200) | 107 (13 – 343) | | 55 (7 – 744) | 229 (21 – 357) | | 146 (7 – 263) |  |
| RAMP3 | Duration (s) | 52 ± 8 | 75 ± 15 | 97 ± 54 | | 65 ± 28 | 38 ± 12 | | 57 ± 14 |  |
|  | Duration per visit (s) | 1 ± 0 | 1 ± 0 | 1 ± 0 | | 1 ± 0 | 1 ± 0 | | 1 ± 0 |  |
|  | Frequency | 61 ± 9 | 78 ± 11 | 47 ± 22 | | 50 ± 20 | 24 ± 7 | | 50 ± 11 |  |
|  | Frequency (%) | 7 ± 1 | 9 ± 1 | 6 ± 2 | | 5 ± 2 | 3 ± 1 | | 4 ± 1 |  |
|  | Latency (s) | 116 (20 – 229) | 85 (16 – 201) | 137 (74 – 1800) | | 58 (12 – 745) | 258 (39 – 358) | | 164 (10 – 403) |  |
| RAMP4 | Duration (s) | 51 ± 11*** | 74 ± 23*** | 25 ± 13 | | 26 ± 18 | 17 ± 9 | | 16 ± 9 |  |
|  | Duration per visit (s) | 2 ± 0 | 2 ± 0 | 1 ± 0 | | 1 ± 0 | 1 ± 0 | | 1 ± 0 |  |
|  | Frequency | 27 ± 5 | 34 ± 6*** | 17 ± 11 | | 12 ± 8 | 8 ± 4 | | 9 ± 4 |  |
|  | Frequency (%) | 3 ± 0 | 4 ± 1** | 2 ± 1 | | 1 ± 1 | 1 ± 0 | | 1 ± 0 |  |
|  | Latency (s) | 117 (33 – 336)** | 115 (27 – 277)* | 1800 (440 – 1800) | | 1039 (273 – 1800) | 1800 (825 – 1800) | | 722 (267 – 1800) |  |
| CIRC | Duration (s) | 19 ± 5 | 12 ± 3 | 104 ± 59 | | 70 ± 19 | 151 ± 48 | | 75 ± 14 |  |
|  | Duration per visit (s) | 1 ± 0* | 1 ± 0 | 2 ± 1 | | 1 ± 0 | 3 ± 2 | | 1 ± 0 |  |
|  | Frequency | 27 ± 6 | 14 ± 3** | 44 ± 10 | | 58 ± 14 | 84 ± 20 | | 79 ± 16 |  |
|  | Frequency (%) | 3 ± 1 | 2 ± 0*** | 6 ± 1 | | 6 ± 1 | 9 ± 1 | | 7 ± 1 |  |
|  | Latency (s) | 138 (75 – 193) | 300 (133 – 369) | 11 (4 – 215) | | 50 (10 – 226) | 32 (18 – 208) | | 29 (0 – 105) |  |
| CENT | Duration (s) | 156 ± 49 | 80 ± 28 | 112 ± 20 | | 104 ± 22 | 138 ± 25 | | 117 ± 15 |  |
|  | Duration per visit (s) | 2 ± 1 | 1 ± 0 | 1 ± 0 | | 1 ± 0 | 1 ± 0 | | 1 ± 0 |  |
|  | Frequency | 83 ± 13 | 46 ± 7* | 130 ± 21 | | 142 ± 28 | 222 ± 37 | | 237 ± 31 |  |
|  | Frequency (%) | 10 ± 2 | 6 ± 1*** | 19 ± 2 | | 17 ± 2 | 24 ± 2 | | 21 ± 2 |  |
|  | Latency (s) | 9 (1 – 30) | 77 (2 – 152) | 3 (1 – 7) | | 3 (0 – 9) | 6 (2 – 18) | | 2 (0 – 40) |  |
| REST | Duration (s) | 148 ± 15 | 140 ± 10 | 121 ± 20 | | 190 ± 27 | 228 ± 49 | | 237 ± 18 |  |

# Numbers represent mean ± SEM except for latency where median median (25% quartile – 75% quartile; the more accurate summarizing statistic for this variable). *p<0.05, **p<0.01, ***p<0.001 relative to AB run 1 animals of the same sex and ^#^p<0.05, ^##^p<0.01 relative to females within the same Strain/Run group.

Abbreviations: CENT, center; CIRC, central circle; CORN, corner; CORR, corridor; DCR, dark corner roof; F, female; M, male; REST, the part of the arena not defined as a zone.

**Supplementary Table 3.** Correlation matrix of activity and duration (s) in the different zones in the zMCSF and novel tank diving test (NTDT) in **(A)** AB zebrafish in Run 1 (upper triangular) and Run 2 (lower triangular) and **(B)** wild zebrafish. Numbers represent statistically significant (α=0.05) Pearson’s correlation coefficients. Numbers represent statistically significant (α=0.05) pair-wise Pearson’s correlation coefficients (*r*) between zones, with positive *r* in green and negative *r* in red shades. Abbreviations: Velocity, velocity in the zMCSF; Totact, total activity in the zMCSF, i.e., sum of all frequencies; START, start zone; DCR, dark corner roof; CORR, corridor; CORN, corner; CIRC, central circle; CENT, center; REST, the part of the arena not designated to any other zone; TOP, top third of the NTDT, MID, middle third of the NTDT, BOT, bottom third of the NTDT.

A

|  | **Velocity** | **Totact** | **START** | **DCR** | **CORR1** | **CORN** | **CORR2** | **RAMP1** | **RAMP2** | **RAMP3** | **RAMP4** | **CIRC** | **CENT** | **REST** | **TOP** | **MID** | **BOT** |
| --- | --- | --- | --- | --- | --- | --- | --- | --- | --- | --- | --- | --- | --- | --- | --- | --- | --- |
| **Velocity** |  | 0.85 |  |  |  | 0.50 |  |  |  |  |  |  |  |  |  |  |  |
| **Totact** | 0.92 |  |  | -0.38 |  | 0.46 |  | 0.43 | 0.57 | 0.52 | 0.37 | 0.44 |  |  |  |  |  |
| **START** |  |  |  |  |  |  |  |  |  |  |  | 0.55 | 0.58 | 0.43 |  | -0.40 |  |
| **DCR** |  |  | -0.46 |  | 0.58 |  |  | -0.45 | -0.65 | -0.71 | -0.47 |  |  | 0.88 |  |  |  |
| **CORR1** |  |  |  |  |  | 0.87 | 0.81 |  |  |  |  | 0.44 | 0.50 | 0.68 |  |  |  |
| **CORN** |  |  |  | -0.41 | 0.85 |  | 0.92 |  |  |  |  | 0.52 | 0.43 | 0.52 |  |  |  |
| **CORR2** |  |  |  | -0.40 | 0.85 | 0.94 |  |  |  |  |  | 0.50 | 0.40 | 0.47 |  |  |  |
| **RAMP1** |  |  |  | -0.61 |  |  |  |  | 0.85 | 0.73 | 0.38 |  |  |  |  |  |  |
| **RAMP2** |  |  |  | -0.54 |  |  |  | 0.81 |  | 0.94 | 0.45 |  |  | -0.52 |  |  |  |
| **RAMP3** |  |  |  | -0.72 |  |  |  | 0.68 | 0.86 |  | 0.64 |  |  | -0.56 |  |  |  |
| **RAMP4** |  |  | 0.37 | -0.47 |  | 0.41 |  |  |  | 0.60 |  |  |  |  |  |  |  |
| **CIRC** |  |  |  |  |  |  |  |  |  |  |  |  | 0.83 |  |  |  |  |
| **CENT** |  | 0.46 |  |  |  |  |  |  |  |  |  | 0.66 |  | 0.57 |  |  | 0.51 |
| **REST** |  |  |  |  |  |  |  |  |  |  |  | -0.48 |  |  |  |  | 0.34 |
| **TOP** |  |  |  |  |  |  |  |  | 0.38 | 0.37 |  |  | -0.44 |  |  | 0.66 | -0.43 |
| **MID** |  |  |  |  |  |  |  |  |  |  |  |  |  |  |  |  | -0.51 |
| **BOT** |  |  |  | 0.58 |  |  |  |  | -0.49 | -0.67 | -0.44 |  |  |  |  |  |  |

B

|  | **Velocity** | **Totact** | **START** | **DCR** | **CORR1** | **CORN** | **CORR2** | **RAMP1** | **RAMP2** | **RAMP3** | **RAMP4** | **CIRC** | **CENT** | **REST** |  |  |  |  |
| --- | --- | --- | --- | --- | --- | --- | --- | --- | --- | --- | --- | --- | --- | --- | --- | --- | --- | --- |
| **Velocity** |  | 0.92 | 0.32 | 0.38 | 0.37 | 0.49 | -0.41 | 0.34 |  | 0.31 | 0.49 |  | -0.44 | 0.47 |  |  |  |  |
| **Totact** |  |  | 0.33 |  | 0.34 | 0.49 | -0.33 | 0.48 | 0.41 | 0.47 | 0.59 |  | -0.36 | 0.44 |  |  |  |  |
| **START** |  |  |  |  |  |  | -0.41 |  |  |  | 0.43 |  |  | 0.53 |  |  |  |  |
| **DCR** |  |  |  |  | 0.56 | 0.43 | -0.36 | -0.31 | -0.32 |  |  |  |  | 0.47 |  |  |  |  |
| **CORR1** |  |  |  |  |  | 0.81 |  |  | -0.35 | -0.36 |  |  |  | 0.59 |  |  |  |  |
| **CORN** |  |  |  |  |  |  |  |  |  |  |  |  |  | 0.56 |  |  |  |  |
| **CORR2** |  |  |  |  |  |  |  |  |  |  |  |  |  | -0.38 |  |  |  |  |
| **RAMP1** |  |  |  |  |  |  |  |  | 0.81 | 0.76 | 0.70 |  | -0.35 |  |  |  |  |  |
| **RAMP2** |  |  |  |  |  |  |  |  |  | 0.93 | 0.71 |  | -0.32 |  |  |  |  |  |
| **RAMP3** |  |  |  |  |  |  |  |  |  |  | 0.86 |  | -0.32 |  |  |  |  |  |
| **RAMP4** |  |  |  |  |  |  |  |  |  |  |  |  | -0.32 |  |  |  |  |  |
| **CIRC** |  |  |  |  |  |  |  |  |  |  |  |  | 0.73 |  |  |  |  |  |
| **CENT** |  |  |  |  |  |  |  |  |  |  |  |  |  |  |  |  |  |  |
| **REST** |  |  |  |  |  |  |  |  |  |  |  |  |  |  |  | **TOP** | **MID** | **BOT** |
|  |  |  |  |  |  |  |  |  |  |  |  |  |  |  |  |  |  |  |
|  |  |  |  |  |  |  |  |  |  |  |  |  |  | **TOP** |  |  | 0.46 | -0.42 |
|  | ***r*** | -1 | -0.8 | -0.6 | -0.4 | -0.2 | 0 | 0.2 | 0.4 | 0.6 | 0.8 | 1 |  | **MID** |  |  |  | -0.31 |
|  |  |  |  |  |  |  |  |  |  |  |  |  |  | **BOT** |  |  |  |  |

**Supplementary Table 4.** Results from the models on the effect of Zone, Run, Sex and their interactions on each response variable in both the zMCSF and novel tank diving test (NTDT). F-values, nominator and denominator degrees of freedom (in subscript) and p-values are presented.

|  |  | zMCSF | | NTDT | |
| --- | --- | --- | --- | --- | --- |
| Distance (cm) | Run | F_1,28_=3.593 | p=0.068 | F_1,28_=2.057 | p=0.163 |
|  | Sex | F_1,28_=7.452 | p=0.011 | F_1,28_=3.453 | p=0.074 |
|  | Run × Sex | F_1,28_=1.007 | p=0.324 | F_1,28_=0.108 | p=0.745 |
| Velocity (cm s^-1^) | Run | F_1,28_=3.614 | p=0.068 | F_1,28_=3.791 | p=0.061 |
|  | Sex | F_1,28_=7.466 | p=0.011 | F_1,28_<0.001 | p=0.990 |
|  | Run × Sex | F_1,28_=1.709 | p=0.322 | F_1,28_=0.090 | p=0.766 |
| Total activity | Run | χ^2^_1,28_=6.648 | p=0.011 | χ^2^_1,28_=3.311 | p=0.096 |
|  | Sex | χ^2^_1,28_=5.098 | p=0.024 | χ^2^_1,28_=0.030 | p=0.797 |
|  | Run × Sex | χ^2^_1,28_=0.052 | p=0.820 | χ^2^_1,28_=3.287 | p=0.080 |
| Duration (s) | Zone | F_11,644_=31.626 | p<0.001 | F_2,168_=16.195 | p<0.001 |
|  | Run | F_1,644_=22.866 | p<0.001 | F_1,168_=1.877 | p=0.173 |
|  | Sex | F_1,28_=0.076 | p=0.785 | F_1,168_=0.002 | p=0.966 |
|  | Zone × Run | F_11,644_=0.425 | p=0.945 | F_2,168_=0.522 | p=0.594 |
|  | Zone × Sex | F_11,644_=0.542 | p=0.875 | F_2,168_=1.974 | p=0.142 |
|  | Run × Sex | F_1,644_=0.105 | p=0.747 | F_1,168_=1.508 | p=0.221 |
|  | Zone × Run × Sex | F_11,644_=0.333 | p=0.968 | F_2,168_=0.446 | p=0.641 |
| Duration per | Zone | F_10,506_=29.676 | p<0.001 | F_2,140_=14.001 | p<0.001 |
| visit (s) | Run | F_1,512_=6.295 | p=0.785 | F_1,140=_7.444 | p=0.007 |
|  | Sex | F_1,29_=9.164 | p<0.001 | F_1,28_=0.672 | p=0.419 |
|  | Zone × Run | F_10,506_=1.013 | p=0.431 | F_2,140_=0.017 | p=0.983 |
|  | Zone × Sex | F_10,506_=1.526 | p=0.126 | F_2,140_=4.875 | p=0.009 |
|  | Run × Sex | F_1,512_=3.273 | p=0.071 | F_1,140_=3.161 | p=0.078 |
|  | Zone × Run × Sex | F_10,506_=0.844 | p=0.587 | F_2,140_=0.744 | p=0.477 |
| Frequency | Zone | F_10,506_=26.109 | p<0.001 | F_2,140_=25.548 | p<0.001 |
|  | Run | F_1,512_=8.150 | p=0.006 | F_1,140=_2.870 | p=0.129 |
|  | Sex | F_1,29_=3.265 | p=0.070 | F_1,28_=0.246 | p=0.600 |
|  | Zone × Run | F_10,506_=0.777 | p=0.731 | F_2,140_=0.325 | p=0.731 |
|  | Zone × Sex | F_10,506_=0.909 | p=0.579 | F_2,140_=0.509 | p=0.604 |
|  | Run × Sex | F_1,512_=0.401 | p=0.568 | F_1,140_=3.086 | p=0.095 |
|  | Zone × Run × Sex | F_10,506_=0.331 | p=0.978 | F_2,140_=0.0132 | p=0.987 |
| Frequency (%) | Zone | F_10,506_=35.485 | p<0.001 | F_2,140_=25.548 | p<0.001 |
|  | Run | F_1,512_=0.003 | p=0.958 | F_1,140=_2.870 | p=0.129 |
|  | Sex | F_1,29_=0.002 | p=0.950 | F_1,28_=0.246 | p=0.600 |
|  | Zone × Run | F_10,506_=0.944 | p=0.489 | F_2,140_=0.325 | p=0.731 |
|  | Zone × Sex | F_10,506_=1.137 | p=0.330 | F_2,140_=0.509 | p=0.604 |
|  | Run × Sex | F_1,512_=0.006 | p=0.939 | F_1,140_=3.086 | p=0.095 |
|  | Zone × Run × Sex | F_10,506_=0.502 | p=0.890 | F_2,140_=0.0132 | p=0.987 |
| Latency (s) | Zone | F_10,588_=28.461 | p<0.001 | F_2,168_=16.884 | p<0.001 |
|  | Run | F_1,588_=2.815 | p=0.094 | F_1,168_=1.929 | p=0.167 |
|  | Sex | F_1,28_=0.005 | p=0.946 | F_1,168_=0.134 | p=0.715 |
|  | Zone × Run | F_10,588_=0.405 | p=0.945 | F_2,168_=0.988 | p=0.374 |
|  | Zone × Sex | F_10,588_=0.915 | p=0.519 | F_2,168_=0.282 | p=0.754 |
|  | Run × Sex | F_1,588_=0.874 | p=0.350 | F_1,168_=0.114 | p=0.736 |
|  | Zone × Run × Sex | F_10,588_=0.585 | p=0.827 | F_2,168_=0.131 | p=0.877 |

**Supplementary Table 5.** Consistency repeatability (i.e. intraclass correlation coefficient or ICC, measured as Pearson’s r) and associated p-value between the first and second run for AB zebrafish of locomotory variables distance moved (cm), velocity (cm s-1) and total activity in the arena as well as zone-specific variables duration (s), duration per visit (s), frequency of zone entries, percentage frequency of zone entries and latency (s) to zone. Abbreviations: CENT, center; CIRC, central circle; CORN, corner; CORR, corridor; DCR, dark corner roof; REST, the part of the arena not designated to any other zone; F, female; M, male.

| **Zone** | **Variable** | **Pearson's *r*** | **p-value** |
| --- | --- | --- | --- |
| WHOLE | Distance (cm) | 0.434 | 0.014 |
| ARENA | Velocity (cm s^-1^) | 0.439 | 0.013 |
|  | Total activity | 0.243 | 0.196 |
| START | Duration (s) | 0.163 | 0.391 |
|  | Duration per visit (s) | 0.050 | 0.794 |
|  | Frequency | 0.122 | 0.522 |
|  | Frequency (%) | 0.066 | 0.731 |
|  | Latency (s) | 0.020 | 0.916 |
| DCR | Duration (s) | 0.358 | 0.052 |
|  | Duration per visit (s) | 0.140 | 0.461 |
|  | Frequency | 0.497 | 0.005 |
|  | Frequency (%) | 0.239 | 0.204 |
|  | Latency (s) | 0.164 | 0.385 |
| CORR1 | Duration (s) | 0.088 | 0.645 |
|  | Duration per visit (s) | 0.096 | 0.614 |
|  | Frequency | 0.214 | 0.257 |
|  | Frequency (%) | 0.253 | 0.177 |
|  | Latency (s) | 0.264 | 0.159 |
| CORN | Duration (s) | -0.044 | 0.819 |
|  | Duration per visit (s) | 0.017 | 0.093 |
|  | Frequency | 0.052 | 0.783 |
|  | Frequency (%) | 0.044 | 0.817 |
|  | Latency (s) | 0.264 | 0.159 |
| CORR2 | Duration (s) | 0.125 | 0.511 |
|  | Duration per visit (s) | 0.556 | 0.001 |
|  | Frequency | 0.129 | 0.498 |
|  | Frequency (%) | 0.098 | 0.607 |
|  | Latency (s) | 0.119 | 0.529 |
| RAMP1 | Duration (s) | 0.508 | 0.038 |
|  | Duration per visit (s) | 0.071 | 0.710 |
|  | Frequency | 0.376 | 0.041 |
|  | Frequency (%) | 0.533 | 0.002 |
|  | Latency (s) | -0.026 | 0.893 |
| RAMP2 | Duration (s) | 0.259 | 0.168 |
|  | Duration per visit (s) | 0.292 | 0.118 |
|  | Frequency | 0.231 | 0.220 |
|  | Frequency (%) | 0.379 | 0.039 |
|  | Latency (s) | -0.066 | 0.727 |
| RAMP3 | Duration (s) | 0.040 | 0.835 |
|  | Duration per visit (s) | -0.080 | 0.676 |
|  | Frequency | 0.052 | 0.784 |
|  | Frequency (%) | 0.127 | 0.505 |
|  | Latency (s) | 0.069 | 0.717 |
| RAMP4 | Duration (s) | -0.033 | 0.861 |
|  | Duration per visit (s) | 0.059 | 0.756 |
|  | Frequency | -0.163 | 0.388 |
|  | Frequency (%) | -0.124 | 0.514 |
|  | Latency (s) | 0.014 | 0.940 |
| CIRC | Duration (s) | 0.156 | 0.410 |
|  | Duration per visit (s) | -0.006 | 0.976 |
|  | Frequency | 0.326 | 0.079 |
|  | Frequency (%) | 0.271 | 0.147 |
|  | Latency (s) | 0.114 | 0.549 |
| CENT | Duration (s) | 0.229 | 0.223 |
|  | Duration per visit (s) | 0.197 | 0.297 |
|  | Frequency | 0.287 | 0.124 |
|  | Frequency (%) | 0.338 | 0.068 |
|  | Latency (s) | -0.097 | 0.612 |
| REST | Duration (s) | 0.133 | 0.485 |

# Supplementary Table 6. Descriptive statistics per experimental group for the novel tank diving test (NTDT). Numbers represent mean ± SEM except for latency where median median (25% quartile – 75% quartile; the more accurate summarizing statistic for this variable). **p<0.01, ***p<0.001 relative to AB run 1 animals of the same sex. Note that this table combines the “strain” and “retested” datasets which were analyzed in two separate models per response variable, see methods section 2.3.

|  |  | **wild run 1** | | **AB run 1** | | | **AB run 2** | | |
| --- | --- | --- | --- | --- | --- | --- | --- | --- | --- |
|  |  | **Females** | **Males** | **Females** | **Males** | **Females** | | **Males** |  |
| ARENA | Sample size | 21 | 22 | 13 | 17 | 13 | | 17 |  |
|  | Distance (cm) | 8924 ± 592*** | 7157 ± 561*** | 4835 ± 516 | 3169 ± 459 | 5588 ± 1206 | | 4369 ± 647 |  |
|  | Velocity (cm s^-1^) | 10.8 ± 0.7** | 8.8 ± 0.6 | 7.2 ± 0.3 | 7.0 ± 0.4 | 8.4 ± 1.3 | | 8.7 ± 0.9 |  |
|  | Total activity | 367 ± 35.8*** | 269 ± 31.4 | 175 ± 19.9 | 230 ± 19.8 | 311 ± 80 | | 238 ± 24.6 |  |
| BOTTOM | Duration (s) | 426 ± 45 | 539 ± 44 | 462 ± 52 | 429 ± 63 | 489 ± 78 | | 392 ± 50 |  |
|  | Visit duration (s) | 8 ± 2 | 9 ± 1 | 8 ± 1 | 6 ± 2 | 12 ± 6 | | 3 ± 0 |  |
|  | Frequency | 86 ± 7^*^ | 79 ± 9 | 63 ± 11 | 91 ± 15 | 129 ± 38 | | 98 ± 13 |  |
|  | Frequency (%) | 26 ± 2 | 32 ± 2 | 36 ± 4 | 41 ± 6 | 40 ± 3 | | 44 ± 4 |  |
| MIDDLE | Duration (s) | 262 ± 23 | 230 ± 25 | 263 ± 28 | 264 ± 41 | 282 ± 65 | | 270 ± 41 |  |
|  | Visit duration (s) | 2 ± 0 | 2 ± 0 | 3 ± 0 | 3 ± 0 | 2 ± 0 | | 2 ± 0 |  |
|  | Frequency | 180 ± 18*** | 131 ± 16 | 78 ± 9 | 88 ± 11 | 133 ± 39 | | 91 ± 13 |  |
|  | Frequency (%) | 49 ± 0 | 48 ± 1 | 44 ± 2 | 38 ± 4 | 42 ± 3 | | 37 ± 3 |  |
| TOP | Duration (s) | 212 ± 28 | 131 ± 22 | 175 ± 33 | 207 ± 40 | 128 ± 36 | | 238 ± 53 |  |
|  | Visit duration (s) | 2 ± 0 | 2 ± 0 | 4 ± 0 | 3 ± 1 | 3 ± 0 | | 4 ± 1 |  |
|  | Frequency | 102 ± 12*** | 58 ± 12 | 35 ± 6 | 51 ± 10 | 49 ± 12 | | 49 ± 13 |  |
|  | Frequency (%) | 25 ± 2 | 20 ± 2 | 20 ± 2 | 21 ± 3 | 18 ± 3 | | 20 ± 3 |  |
|  |  |  |  |  |  |  | |  |  |

# Supplementary Figures

A

B

C

D

**Supplementary Figure 1.** Blueprints of the zMCSF. Measurements in millimeters (mm) of the zMCSF arena and the different components. **(A)** cubic shaped empty arena; **(B)** dark corner roof (DCR); **(C)** walls creating the corridor zones, made of infrared transparent plastic; **(D)** inclined plane of the ramp, with attached wall to rule out a direct pathway to the top of the ramp. Placement of parts B-D is described in Figure 1.

A

B

**Supplementary Figure 2.** Principal component analysis (PCA) of the behavioral variables from the zMCSF for AB zebrafish in Run 1 and 2. Scatterplots of **(A)** individual scores on PC2 vs. PC1 (score plot) and **(B)** variable loadings on PC2 against PC1 (loading plot). Abbreviations: CENT, center; CIRC, central circle; CORN, corner; CORR, corridor; DCR, dark corner roof; REST, the part of the arena not designated to any other zone; Dur, duration; Durfreq, average duration per visit; Freq, frequency; Freqperc, percentage frequency; V, mean velocity in the arena.

A

B

**Supplementary Figure 3.** Behavior in the zMCSF over testing time (in minutes). **(A)** Duration (s) in the different zones over time. **(B)** Frequency of zone entries in the different zones over time. Colored bars represent the mean per strain/sex/run group (from top to bottom): wild females and wild males, AB females run 1, AB males run 1, AB females run 2, AB males run 2. Abbreviations: CENT, center; CIRC, central circle; CORN, corner; CORR, corridor; DCR, dark corner roof; REST, the part of the arena not designated to any other zone; F, female; M, male.
